# Adaptive Journeys: Accelerating Cross-Cultural Adaptation Through Study Tours

**DOI:** 10.3390/bs15070973

**Published:** 2025-07-17

**Authors:** Ziye Huang, Anmin Huang, Ziyan Yin

**Affiliations:** College of Tourism, Huaqiao University, Quanzhou 362021, China; ziye_huang@stu.hqu.edu.cn (Z.H.); 17013121019@stu.hqu.edu.cn (Z.Y.)

**Keywords:** study tours, cross-cultural adaptation, environmental settings, situated learning theory, embodied cognition theory

## Abstract

With the rise in short-term intercultural mobility programs, enhancing students’ adaptive capacity through structured experiential learning has become a key concern. Using constructivist grounded theory, this study draws on extending the situated learning and embodied cognition theories as analytical frameworks to explore international students’ cross-cultural adaptation within the context of study tours. It develops a three-phase framework (pre-departure, on-site immersion, and post-tour reflection) to trace their transition from cultural distance to adaptation. The findings reveal that the process through which international students shift from cross-cultural distance to multidimensional adaptation can be further accelerated by environmental settings, situational behaviors, and short-term emotional responses within study tour contexts. Moreover, culture-led and nature-led environments evoke distinct patterns of participation and emotional responses, facilitating varying degrees and dimensions of adaptation across psychological, social, and cultural domains. The study extends situated and embodied learning perspectives by conceptualizing study tours as dynamic, context-sensitive learning sites. By considering adaptation processes context-dependent, this study deepens the understanding of how learning, emotion, and environment interact to shape intercultural development and offers practical insights for designing responsive, stage-sensitive study tour programs.

## 1. Introduction

In today’s globalized education landscape, over 6.4 million students studied abroad in 2021 (UNESCO Institute for Statistics (UIS), 2023). While international mobility creates opportunities for intercultural learning and global engagement, students often face cultural barriers—such as language difficulties, unfamiliar norms, and social disconnection—that hinder emotional well-being and adaptation (Andrade, 2006; Osler & Zahavi, 2023). These challenges are especially acute in post-COVID-19 contexts, where digital isolation has intensified cultural alienation.

Traditional classroom-based approaches to intercultural education often fail to support students’ deeper emotional and behavioral adjustment (McGladdery & Lubbe, 2017b). In contrast, study tours—short-term, university-organized cultural programs—offer immersive, experiential exposure to local settings that encourage direct cultural participation and peer interaction. As non-curricular but structured experiences, they provide students with embodied engagement and real-world cultural encounters (Iskhakova & Bradly, 2022), fostering intercultural empathy, resilience, and social belonging (Alsubaie, 2015).

Despite their growing use, most empirical studies on study tours remain outcome-focused, emphasizing learning gains or satisfaction levels (Gebhard, 2012), with limited insight into the transformation mechanisms during the tours. Moreover, dominant models of cross-cultural adaptation tend to be linear or typological, overlooking how adaptation unfolds in situated, sensory-rich, and relational contexts (Karim, 2021).

To address this gap, the study introduces the situated learning theory (Lave & Wenger-Trayner, 1991/2011) and embodied cognition theory (Wilson & Beard, 2013), conceptualizing study tours as embodied and situated learning spaces and develops a processual framework to explore how study tours, as a process variable, accelerate international students’ transition from cross-cultural distance to adaptation. Three main questions were aimed to be solved: (1) what are the processual pathways and dimensions through which international students transition from cross-cultural distance to adaptation catalyzed by study tours; (2) how do study tours, as situated and embodied learning spaces, facilitate this transition, and through what mechanisms; (3) how do different environment-led factors differentially impact the outcomes of cross-cultural adaptation. By addressing these questions, this study aims to offer a new perspective on short-term intercultural learning and contribute to a deeper understanding of how international mobility fosters meaningful cross-cultural adaptation beyond the classroom.

## 2. Literature Review

### 2.1. Study Tours in the Context of Cross-Cultural Experience

The study tour concept has evolved from the aristocratic European Grand Tour (Scholten et al., 2022) into a widespread form of experiential learning in intercultural education. Modern study tours provide structured, short-term exposure to local cultures through direct participation rather than passive observation (Pitman et al., 2010). These programs, often organized by educational institutions, immerse students in authentic social and cultural contexts, enabling them to engage with local customs and communities, build intercultural awareness, and begin identity negotiation processes.

Research confirms the educational and emotional benefits of study tours. Early studies report cultural intelligence and intercultural sensitivity improvements by study tours (Young & Schartner, 2014; O’Reilly et al., 2014). Recent work highlights their potential to foster empathy, self-reflection, and identity development in unfamiliar environments (McGladdery & Lubbe, 2017a; Vieira et al., 2022). However, the literature remains outcome-driven, mainly focusing on learning gains or satisfaction, with limited exploration of these transformations’ underlying processes.

Some scholars address barriers international students face, such as language and psychological challenges (Gong et al., 2021; Chaiyasat, 2020), yet fail to link these challenges to study tours’ embodied or emotional dynamics. Others explore cultural immersion through peer interaction (Chwialkowska, 2020), leisure learning (Zhang et al., 2022), or immersive travel (Nelson & Luetz, 2021), but often conflate study tours with broader mobility programs or tourism. Importantly, most of these studies focus on Western host countries, overlooking how international students adapt in non-Western cultural systems like China.

What remains underexplored is how study tours, as structured but non-curricular experiential environments, activate specific mechanisms—especially embodied and emotional ones—that contribute to adaptation. This study addresses that gap by examining how cultural and natural settings within study tours support cultural integration, social inclusion, and psychological adjustment among international students in China.

### 2.2. Cross-Cultural Adaptation: From Classic Models to New Perspectives

Cross-cultural adaptation refers to how individuals adjust to new cultural environments, reshaping their behaviors, emotions, and identities (Berry, 1997). Foundational theories have shaped our understanding of this process, including Oberg’s (1960) culture shock model, Searle and Ward’s (1990) dual model of psychological and sociocultural adaptation, and Berry’s (1997, 2005) acculturation framework.

Oberg’s model outlines four stages of emotional adjustment—honeymoon, crisis, recovery, and adjustment—but has been criticized for oversimplifying adaptation as a linear process. Searle and Ward (1990) introduced a more nuanced distinction between psychological (well-being) and sociocultural (social competence) dimensions. Berry (1997) proposed four strategies—assimilation, separation, integration, and marginalization—emphasizing integration as the most favorable. Yet, these models are often seen as overly static, ignoring how individuals flexibly shift between strategies depending on context (Karim, 2021).

Recent studies increasingly recognize that adaptation is not merely cognitive or rational, but also embodied, emotional, and situational (Sozen, 2020; Riaz et al., 2023). These newer perspectives stress that cultural learning unfolds through situational, sensuous, and affective experiences, particularly in immersive, short-term settings like study tours. However, few studies have systematically integrated these components into broader theories of adaptation.

This study builds on these critiques by proposing a process-oriented view of adaptation. Rather than a fixed sequence, adaptation is conceptualized as a dynamic, meaning-making process that is accelerated and shaped through physical engagement, sensory immersion, cognitive exchange, and social bonding within the situated context of the study tour.

### 2.3. Situated Learning and Embodied Cognition Theories in Study Tours

The situated learning theory, proposed by Lave and Wenger-Trayner (1991/2011), posits that learning occurs most effectively within authentic, socially and culturally situated environments. Rather than abstract transmission, knowledge and adaptation are seen as emerging from active participation in meaningful practices. In the context of study tours, international students are removed from conventional classroom settings and immersed in real-world, context-rich environments where they interact with local people, navigate social norms, and engage in culturally embedded activities. While the theory has been widely applied in the field of education (O’Brien & Battista, 2020), application to tourism and cross-cultural learning is still underexplored. However, its core concepts—learning as social participation, knowledge as situated, and identity as relational—provide a strong theoretical foundation for understanding how international students undergo cultural adjustment during study tours.

The embodied cognition theory (Lakoff & Johnson, 1980) complements this perspective by emphasizing the role of the body in shaping thought, perception, and emotion. Physical experiences and interactions shape perception, memory, and emotion. It posits that cognition is not confined to the brain but grounded in physical and sensory engagement with the environment. This is particularly relevant in study tours, where students’ learning is closely tied to sensory immersion, such as tasting unfamiliar foods, walking in natural landscapes, or participating in traditional cultural practices. Embodied experiences such as these do not merely transmit information but evoke emotional shifts and bodily memories that support meaning-making, identity negotiation, and adaptation (Glenberg, 2010; Li et al., 2024; Grimes-MacLellan, 2005).

The integration of these two perspectives aligns closely with this study’s objectives: to explore how international students transition from cross-cultural distance to adaptation through embodied, situated encounters during study tours. The situated learning theory helps explain the contextual and relational nature of adaptation, while embodied cognition highlights how sensory and physical engagements catalyze emotional and cognitive transformation. Together, these frameworks allow for a more holistic understanding of how short-term mobility programs function as academic enrichment and as immersive, adaptive experiences shaped by space, body, and emotion.

## 3. Materials and Methods

### 3.1. Method Selections

Due to varying schools of grounded theory, we clarify our rationale for adopting constructivist grounded theory (CGT) in this study. Our research aims to uncover the processual mechanisms through which study tours catalyze international students’ cross-cultural adaptation—shape international students’ cross-cultural adaptation—an area where existing studies remain largely outcome-oriented and under-theorized (Martin & Woodside, 2008; Zuo et al., 2022). Grounded theory is particularly well-suited for theory-building in such domains, as it facilitates the inductive generation of conceptual frameworks grounded in lived experience (Matteucci & Gnoth, 2017).

Among the various grounded theory traditions, the original formulation by Glaser and Strauss (1967) and the proceduralized model by Strauss and Corbin (2003) assume that theory is “discovered” from data, implying that data exist independently of the researcher. However, this objectivist stance overlooks the interpretive nature of qualitative inquiry. Prior to adopting CGT, we conducted a pilot test in which three researchers independently coded the same qualitative dataset. Despite following a shared protocol, the coders’ results consistently differed, underscoring that individual perspectives shape data interpretation and cannot be entirely objective.

This epistemological insight aligns with CGT’s premise that data are co-constructed through researcher–participant interaction (Charmaz, 1995, 2006). CGT thus acknowledges the active role of the researcher while retaining a commitment to systematic rigor. Furthermore, it supports our view that cross-cultural adaptation is a context-sensitive, meaning-making process shaped by embodied experience. This perspective resonates with our theoretical framing, which integrates situated learning and embodied cognition, emphasizing the dynamic interplay between behavior, environment, and interpretation. CGT, therefore, offers both the philosophical grounding and methodological flexibility needed to examine how adaptation unfolds within the unique experiential contexts of study tours (Charmaz & Thornberg, 2021).

### 3.2. Data Collection and Sampling

Data were collected at a Chinese university with a longstanding international focus, enrolling nearly 10,000 students from over 90 countries per year. This culturally diverse environment provides a rich empirical setting to examine patterns of adaptation. All the participants in this study were international students pursuing a degree or language programs at the university. Their diverse national and cultural backgrounds, combined with sustained exposure to the local academic and social environment, made them particularly well-suited to provide insights into the process of cross-cultural adaptation in the context of study tours. They are designed to foster intercultural understanding by bringing international students to culturally and ecologically significant destinations across China. Each tour typically spans 3–5 days and includes curated activities such as museum visits, traditional craft workshops, and immersive experiences in scenic natural environments—such as mountain areas and coastal sites. These activities are intentionally designed to facilitate experiential learning, emotional resonance, and social bonding beyond the classroom. Such tours are considered effective mechanisms for cross-cultural adaptation because they provide students with multisensory, embodied encounters with local culture in socially embedded settings. Unlike routine classroom study, study tours stimulate contextual engagement and interaction, which prior research suggests are critical for developing cultural understanding, reducing anxiety, and building social inclusion (Leung et al., 2011; Buckley, 2020). This aligns with the situational learning theory, where adaptation is seen as a process emerging from situated and embodied learning. Accordingly, we treat the study tour not only as a situational site that actively initiates and accelerates intercultural development.

Study Tour Diaries: A total of 101 study tour reflection diaries were initially collected from the university’s online platform between 2019 and 2022. These diaries were written in response to structured post-tour prompts as part of a reflective learning assignment to encourage students to articulate their intercultural experiences, emotional reactions, and personal growth. Each diary typically consisted of 900–1500 words and was completed after the study tour. Initially, theoretical saturation was reached through the analysis of 78 study tour diaries, which captured students’ experiences across different phases of the study tour. However, given that most diaries were written within a month after the tours, the depth of longitudinal reflection was limited.

Semi-Structured Interviews: The main purpose of the interviews was to deepen and supplement the theory dimension of post-tour reflections, which were relatively thin in diaries due to the short time interval between participation in study tours and writing down diaries. Thus, we conducted semi-structured interviews with international students who had participated in study tours for more than half a year, allowing us to explore longer-term meanings and adaptive impacts of the study tour experience. Interview questions were designed a preliminary analysis of the diary data, the key domains included memory of intercultural encounters, physical and emotional responses to study tour activities, reflections after study tours, and perceived changes in cross-cultural adaptation over time (see Appendix A). Participants were selected through purposive sampling to ensure diversity in gender, nationality, academic discipline, and length of stay in China. While we recognize that variation in the duration of residence—from several months to over three years—may lead to differences in adaptation outcomes, this diversity was intentionally preserved to enrich the dimensionality of our findings. Instead of treating this variability as a confounding factor, we regarded it as a context-sensitive variable that allows for meaningful comparison across different phases or levels of cross-cultural adaptation, such as distinguishing surface cultural awareness from deeper identity formation or sustained social engagement. This decision is further addressed in the limitations section, where we reflect on how such heterogeneity both enhances and complicates interpretation. Recruitment was conducted via email invitations and snowball sampling among former tour participants. The interviews (30–50 min) followed a flexible protocol that allowed participants to elaborate on experiences related to themes emerging from the diaries. Table 1 shows the basic information of the interview participants.

Together, theoretical saturation was approached by the fourth interview, after which a theoretical saturation check was conducted through two additional interview transcripts and the remaining 23 diaries. No new conceptual categories emerged, confirming the sufficiency and robustness of the data.

### 3.3. Data Analysis

Data analysis followed Charmaz’s (2006) CGT approach and proceeded iteratively (Lindqvist & Forsberg, 2023). Three researchers with tourism and qualitative research backgrounds independently coded a subsample of 78 diaries to ensure interpretive reliability, followed by collaborative reconciliation. NVivo 12.0 facilitated data organization and comparison.

Initial coding involved breaking down data into smaller meaning units, yielding 311 codes. These codes were subsequently grouped into 52 initial concepts (Matteucci & Gnoth, 2017). Labels and memos were used to summarize initial codes and record analytical insights, facilitating concept development and theory building. Table 2 provides examples of initial coding from raw data.

During focused coding, initial codes were consolidated into sub-categories and grouped under broader themes such as cross-cultural distance, emotional responses, and situational behaviors. This stage involved identifying the most salient and recurring codes from the initial round and comparing them across different data entries to establish interpretive consistency (Charmaz, 2006). Through constant comparison, we began to detect patterns in how students responded to different environments and situations. In particular, we observed that many diary excerpts described students’ embodied interactions with the physical environment, which led to the emergence of categories such as physical engagement, sensory immersion, cognitive exchange, and social bonding. The situated learning theory theoretically informed the categorization of these situational behaviors (Lave & Wenger-Trayner, 1991/2011), which emphasizes how knowledge is constructed through social participation in context-specific settings. At the same time, the embodied cognition theory (Wilson & Beard, 2013) guided our interpretation of sensory and affective expressions in the data, allowing us to conceptualize embodied experiences as meaningful triggers of emotional and adaptive responses. This phase helped reduce data complexity and laid the groundwork for higher-level abstraction in axial coding.

During the axial coding process, as the analysis of the diary data deepened, we gradually identified several recurring mid-level themes about cross-cultural adaptation outcomes, such as cultural participation, social bonding, and emotional stability. Initially, we attempted to interpret these patterns using classic cross-cultural adaptation models (Searle & Ward, 1990; Berry, 1997). While these models provided a foundational framework for understanding general acculturation modes, they proved insufficient in capturing the nuanced and multi-dimensional transformations evident in students’ post-tour reflections, particularly regarding how “integration” manifested across different domains. Thus, we supplemented the interview material and categorized cross-cultural adaptation into three dimensions—cultural, social, and psychological—and further delineated three levels of adaptation within each dimension.

**Figure 1:**
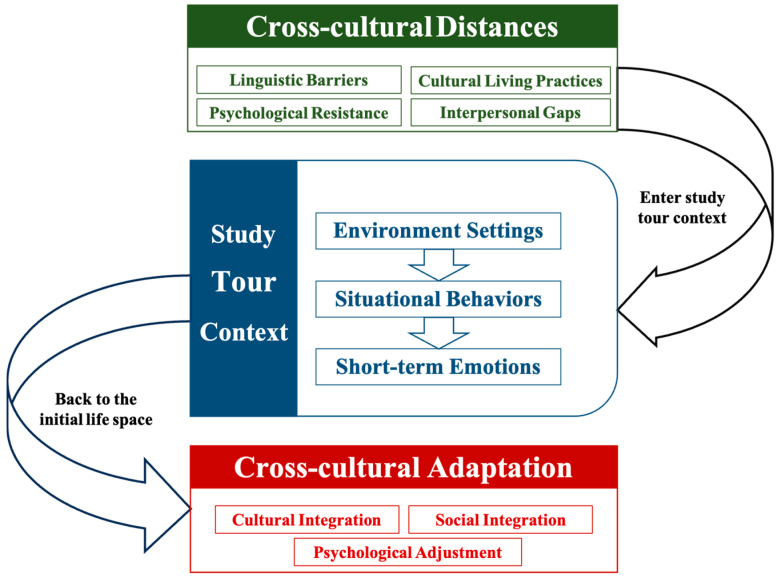
Diagram of the main categories’ relationship structure.

To better capture the evolving nature of students’ experiences across the pre-tour, on-site, and post-tour phases, theoretical coding was guided by a processual framework proposed by Porth’s (1997) three-phase strategic model. This framework enabled a more systematic mapping of the temporal trajectory of cross-cultural adaptation. It helped integrate key categories into a cohesive explanation of how study tours catalyze adaptation (see Figure 1).

## 4. Results

This study reveals a dynamic process in which international students shift from experiencing cross-cultural distance to achieving cross-cultural adaptation. Through CGT, a conceptual structure was developed to capture this transformation, encompassing cross-cultural distance, situational behaviors, short-term emotional responses, and multiple dimensions of adaptation (see Table 3).

Initially, in traditional classroom settings and daily life, international students often encounter multiple cross-cultural distances—ranging from linguistic difficulties and unfamiliar cultural norms to psychological discomfort and interpersonal gaps—that hinder their ability to adapt effectively. These barriers tend to create emotional detachment and limit opportunities for meaningful engagement with the host environment.

During study tours, however, students enter a situational and embodied learning environment that triggers distinct situational behaviors and emotional responses, depending on the environmental settings. Four key types of situational behaviors were identified: physical engagement, sensory immersion, cognitive exchange, and social bonding, each accompanied by cognitive emotions (e.g., curiosity or pride) or affective states (e.g., calmness or joy).

**Figure 2:**
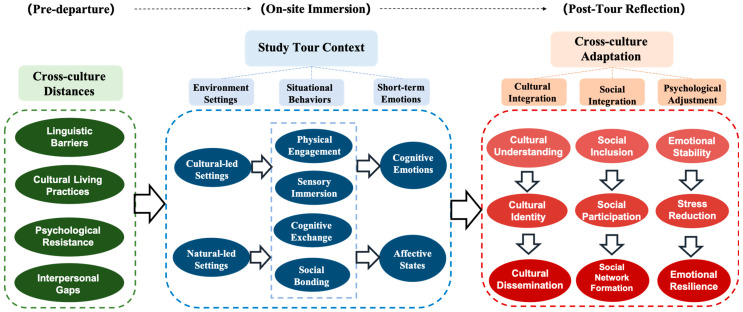
Model of the study tour catalyzes international students from cross-cultural distance to adaptation.

As students internalized these affective and embodied moments post-tour, many reported greater openness, increased confidence, and a strengthened sense of belonging, marking their progression toward deeper cross-cultural adaptation. Notably, different environmental settings elicit distinct emotional-behavioral dynamics and thereby elicit distinct cross-cultural adaptation dimensions: culture-led environments primarily facilitated cultural integration and identity formation, while nature-led settings promoted psychological adjustment and social integration. The overall transformation process—linking pre-departure, on-site immersion, and post-tour reflection—is illustrated in Figure 2. The following sections elaborate on the specific mechanisms and categories underpinning this transformation.

### 4.1. Pre-Departure: Identifying Cross-Cultural Distance

Before participating in study tours, international students often face multiple cross-cultural barriers in both academic and everyday contexts. These barriers typically fall into four interrelated dimensions: linguistic barriers, cultural living practices, psychological resistance, and interpersonal gaps. Among them, linguistic barriers refer to limited language proficiency, which impedes both understanding and expression. This often leads to frustration and withdrawal in social settings. For instance, a student who had just arrived in China described the following:

*“I was not able to communicate well with others usually because I could not speak English or Chinese very well, and I often felt disappointed.”*

(D33)

This illustrates how communication difficulties can become a source of self-doubt, discouraging social engagement.

Cultural living practices reflect the disconnect students feel when encountering unfamiliar daily routines. A student wrote the following:

*“In the morning, we had breakfast in the cafeteria. Although some of us were not used to it, I’m sure we’ll all love Chinese breakfast later.”*

(D67)

While the tone here suggests openness, the initial discomfort underscores the subtle disorientation caused by unfamiliar food and customs in early adaptation.

Psychological resistance manifests as feelings of marginality or hesitation when engaging with the host culture. Students sometimes referred to local norms from a distance using generalized third-person phrasing:

*“I heard that Chinese people usually use tea to entertain guests”*

(D39)

Such expressions suggest limited identification and lingering cultural alienation during the early phase.

Interpersonal gaps emerge from unfamiliarity with local social norms and a lack of emotional closeness. One participant initially felt hesitant about interacting with others, especially those perceived as culturally distant:

*“I was able to talk to my friends from the Dutch and German teams. I thought it would be difficult to interact with people from other countries, but everyone was very nice!”*

(V6)

Here, preconceived notions gave way to unexpected positive interactions, but the initial social distance highlights a key barrier to adaptation.

Together, these four dimensions characterize the pre-tour cultural distance faced by international students. They set the stage for how study tours may later function as catalysts for meaningful adaptation.

### 4.2. On-Site Immersion: The Study Tour as a Catalyst for Adaptation

During the study tour, international students enter an embodied and interactive learning environment that supports their transition from cultural distance to adaptation. Drawing on Lave and Wenger-Trayner’s (1991/2011) situated learning framework, we conceptualize this phase as the “study tour situation,” shaped by three interlinked elements: environmental settings, situational behaviors, and short-term emotions.

#### 4.2.1. Environment Settings

The physical and social environments of the study tour form the structural basis for experiential learning. Two primary types emerged from the data: culture-led settings (e.g., heritage sites, workshops, and factory visits) and nature-led settings (e.g., mountains, beaches, and parks). While many study tours involved a combination of both, students’ reflective narratives typically delineated these two types of settings clearly, often emphasizing distinct aspects of experience associated with each. For instance, one participant recounted the following:

*“After arriving in Huangshan, the first trip was to Huizhou’s famous ink manufacturing place, where we watched how traditional ink is made and even tried grinding the ink ourselves. I was amazed at the precision and patience required—it gave me a new appreciation for Chinese calligraphy… The next day, climbing the Yellow Mountain and breathing the fresh air, I suddenly felt calm and deeply connected to nature. It was unforgettable.”*

(D16)

Although this itinerary involved both cultural and natural elements, the student’s reflection clearly distinguished between the structured, knowledge-based learning at the ink factory and the emotionally rich, immersive experience during the mountain climb. Such contrastive reflection supports our decision to analytically separate these two types of settings, as they tend to foster distinct experiential modes that contribute to different facets of cross-cultural adaptation.

#### 4.2.2. Situational Behaviors

The embodied cognition theory (Lakoff & Johnson, 1980; Wilson & Beard, 2013) informed the analysis of students’ behavioral responses during the study tour, highlighting how bodily and sensory experiences contribute to adaptation. Four situational behaviors through which students actively or passively engage with their surroundings were identified: physical engagement, sensory immersion, cognitive exchange, and social bonding.

**Physical engagement** refers to students’ active, bodily participation in activities, such as hands-on crafting, hiking, or food making. Unlike passive observation, these actions required intentional involvement and allowed students to experience the culture through movement and touch:

*“I even colored the top of the ink myself.”*

(D19)

This quote demonstrates proactive involvement, which lays the groundwork for a deeper cultural connection and potential emotional resonance.

**Sensory immersion**, by contrast, involves passive yet impactful environmental experiences, such as sights, sounds, and smells. In culture-led settings, it often centers on visual and auditory inputs—exhibits, performances, or folk crafts:

*“After listening to the tour guide, I realized the jade is valuable!”*

(D18)

Nature-led settings engage a broader range of senses, offering rich stimuli such as birdsong, ocean sunrises, fresh air, and gentle breezes:

*“The scenery from there was stunning, and you could finally see China’s great mountains and rivers. The air there was so fresh, with the fragrance of leaves and birdsong all along the road, exactly like Europe!”*

(D66)

Such immersive exposure helps students develop a bodily familiarity with the host environment, preparing them for deeper emotional openness.

**Cognitive exchange** refers to dialogic or observational encounters that stimulate reflection and enhance cultural understanding. These include guided explanations, interactions with locals, or structured learning moments that encourage intellectual engagement:

*“Under the teacher’s language guidance and action correction, I learned the basic skills—seven steps and three stops.”*

(D8)

Such interactions often triggered moments of curiosity or insight, priming students to interpret cultural differences not as barriers, but as meaningful learning opportunities.

**Social Bonding** involves interpersonal connections formed through shared experiences, collaboration, or informal interactions. This behavior includes peer support during group travel and spontaneous exchanges with locals. In nature-led contexts, bonding often occurred through collective leisure or physical activity:

*“Everyone is pleased today. It is also kind of nice to speak with the Netherlands and Germany team buddies; I thought the other countries were challenging to be my friends, but everyone is very nice! It is memorable to climb together with the class, I hope that the next few days everyone can be as happy as today”*

(D68)

In culture-led settings, connections emerged during communal tasks and hospitality encounters:

*“The uncles, aunts, and children there were very enthusiastic. Each of us learned to make cakes seriously. Although I didn’t get to experience it, an adorable kid gave me the cake she made!”*

(D49)

These moments of bonding not only reduced feelings of cultural alienation but also created an emotional bridge toward trust and belonging, conditions necessary for deeper adaptation.

#### 4.2.3. Short-Term Emotions

Short-term emotions refer to the immediate affective responses triggered by students’ interactions with people, environments, and activities during the study tour. While transient, these emotions serve as key psychological mechanisms that initiate and sustain the process of cross-cultural adaptation. Based on our analysis, these emotional reactions can be grouped into two categories: cognitive emotions and affective states.

Cognitive emotions are evaluative in nature, often sparked by moments of insight, curiosity, or personal achievement. These emotions not only heighten students’ intellectual engagement with the host culture but also contribute to a sense of purpose and meaning in their experiences. For example, one student described a moment of pride and accomplishment after completing a cultural task:

*“The moment my self-created paper weaving painting was framed, my heart was filled with a sense of fulfillment”*

(D9)

This illustrates how hands-on participation can generate a sense of ownership and recognition, reinforcing students’ cultural confidence and motivating further engagement.

In contrast, affective states refer to more diffuse, mood-based feelings such as relaxation, calmness, joy, or emotional relief. These are typically evoked by sensory immersion or social bonding in low-pressure, natural settings. For instance, one student reflected on the emotional comfort and joy experienced during group travel:

*“With the company of my companions, the journey was not tiring at all and was even very pleasant and joyful.”*

(D61)

Such emotional states, though short-lived, play a critical role in reducing cultural anxiety and creating openness to new environments. They act as emotional anchors that help students feel safe and receptive, especially in the early stages of intercultural adaptation.

### 4.3. Post-Tour Reflection: Bridging Experiences to Cross-Cultural Adaptation

The post-tour phase reflects the consolidation of learning and the beginning of deeper intercultural adjustment. As students return from the tour and re-enter everyday academic life, they begin to internalize their embodied and emotional experiences, transforming them into more stable cognitive, emotional, and behavioral patterns. This phase is characterized by three interrelated adaptation outcomes: cultural integration, social integration, and psychological adjustment.

#### 4.3.1. Cultural Integration

Cultural integration describes how students move from initial observation to affective identification and eventually to active cultural sharing. These layers reflect an expanding engagement with the host culture:

Cultural understanding involves an emerging awareness of local customs and values. Students begin to articulate these observations with curiosity and respect, often in a descriptive tone:

*“I found that people in Wuyishan love tea.”*

(D63)

Cultural identity develops when students begin to connect emotionally with cultural practices and express a desire for preservation or continuity. This signals a shift from outsider observation to personal resonance:

*“I love these cultures so much that whenever I visit a place that utilizes handmade items, I feel nothing but admiration and a secret hope that they will pass on these crafts, and I don’t want my future generations to see and feel nothing but cold, machine-made products.”*

(D17)

Cultural dissemination is an advanced level of cultural engagement, where students act as informal cultural ambassadors, recontextualizing what they learned for new audiences in their home countries:

*“I am very interested in the Chinese tea art. I visited the site, learned about the tea-making process from the lecturer, and recorded a video explanation in Filipino in the workshop. I plan to share the results of my study tour with Filipino primary and secondary school students in class when I return to my home country.”*

(D35)

These shifts suggest that study tours can facilitate not just understanding, but also meaningful cultural alignment and agency.

#### 4.3.2. Social Integration

Social integration reflects how students build a sense of belonging and forge interpersonal ties that extend beyond the tour. It evolves across three levels:

Social inclusion occurs when students feel welcomed and emotionally supported by host community members. These positive social signals help reduce perceived barriers:

*“Thanks to Ms. Liu and Ms. Chen for their care and help during these three days, and I feel very close to them!”*

(D53)

Social participation emerges when students actively seek out opportunities to re-engage with local events or initiatives, extending the tour’s influence into campus life:

*“I plan to participate in the study activities organized by the college in the future because I can learn much knowledge from these activities that is not in the textbooks.”*

(D53)

Social network formation captures the creation of longer-term friendships that serve as support systems for continued adaptation:

*“I also made a friend named Zhang Jing at that time. We still hang out together now.”*

(V4)

These developments indicate that the study tour served not only as a site of temporary connection but also as a meaningful springboard for sustained social embeddedness in the host environment.

#### 4.3.3. Psychological Adjustment

Psychological adjustment denotes the emotional self-regulation and personal growth that emerge from having overcome earlier cultural challenges. It comprises the following:

Emotional stability, marked by greater ease and confidence in navigating daily life in the host country:

*“I started not to be afraid to socialize with local students… Now, I feel happy with my life of studying abroad!”*.

(V2)

Stress reduction, reflecting the fading of initial anxiety and increased willingness to seek help or clarification:

*“Even if I still cannot understand clearly sometimes, I will ask the teacher after class… every teacher treats us with great patience.”*

(V1)

Emotional resilience, defined by students’ perceived growth in adaptability and confidence to face future intercultural challenges:

*“Participating in the study tour is like a door, opening the way for me to become confident and comfortable… I even believe I will do better in any country I go to in the future.”*

(V1)

These psychological outcomes suggest that the study tour experience played a vital role in fostering emotional resilience and empowering students to navigate future intercultural challenges with greater confidence.

### 4.4. How Different Environmental Settings Shape Cross-Cultural Adaptation

**Figure 3:**
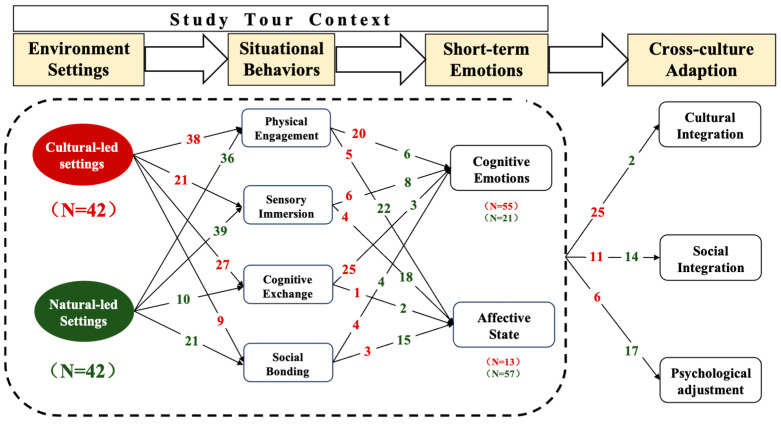
Types and frequency of different situational behaviors, short-term emotions, and cross-cultural adaptation triggered by cultural and natural environment settings (unit: times). Note: the red numbers represent the number of times the culture-led texts mention the corresponding situational behaviors, short-term emotions, and cross-cultural adaptation, and the green numbers represent the number of times the nature-led texts mention the corresponding situational behaviors, short-term emotions, and cross-cultural adaptation.

To deeply unpack how different environmental contexts shape the adaptation outcome of international students, this study conducts a further comparative analysis of culture-led and nature-led environments. These settings exhibit distinct affordances in eliciting situational behaviors, short-term emotions, and ultimately, different dimensions of cross-cultural adaptation. Based on the textual coding of the diary and interview excerpts (42 from each setting, with the culture-led group randomly sampled from a pool of 61), a differentiated pattern of engagement and emotional activation emerged (see Figure 3).

**Culture-led environments**, such as heritage sites, workshops, and instructor-guided sessions, were characterized by structured, cognitively rich activities. These contexts often involved hands-on bodily engagement (e.g., handicrafts or traditional food-making) and explicit knowledge exchange (e.g., guided explanations or Q&A with local experts), stimulating cognitive emotions such as curiosity, pride, and admiration:

*“When I successfully made a bracelet, I really felt proud”*

(D22)

Such symbolically dense interactions promote cultural meaning-making and identity negotiation. These settings are thus more strongly associated with outcomes in cultural integration, including identification with local values and a desire to disseminate host culture knowledge:

*“After learning how to make the paper-cutting patterns, I strongly connected to the stories behind them. It wasn’t just fun—it made me proud to be part of this. I’ve already prepared a video to introduce it in my school back home.”*

(D21)

**Nature-led environments**, such as mountains, beaches, and parks, by contrast, offer low-pressure, sensory-rich experiences that encourage physical movement (e.g., hiking) and spontaneous peer interactions. These settings amplify affective states such as calmness, joy, and emotional release:

*“Chatting with newly made friends by the seaside… I felt an unprecedented sense of relaxation and happiness.”*

(D31)

This embodied and emotional engagement pathway contributes more directly to psychological adjustment (e.g., stress relief or emotional resilience) and social integration (e.g., bonding or inclusion), especially in the early phases of adaptation when cultural unfamiliarity and emotional anxiety are high:

*“Walking together by the lake and laughing with my classmates made me feel relaxed and connected—it was the first time I didn’t feel like a stranger here.”*

(D55)

While this study differentiates between culture-led and nature-led settings for analytical clarity, this distinction serves primarily as a heuristic device. In practice, experiences involve blended elements—for example, a tea-making activity in a mountain village may simultaneously offer cultural instruction and natural immersion. We do not suggest that these categories are mutually exclusive; rather, separating them helps to better identify the dominant experiential mode and examine how each type may differentially elicit emotional responses, situational behaviors, and ultimately, pathways of cross-cultural adaptation. Typically, culture-led settings emphasize structured participation and cognitive engagement, which foster cultural identity, cultural dissemination, and a sense of social inclusion. In contrast, nature-led settings tend to evoke affective and sensory immersion, which support psychological adjustment and social bonding with peers.

In sum, the juxtaposition of environmental triggers and behavioral responses illustrates the processual and context-contingent nature of cross-cultural adaptation. These findings highlight how mobility-based education can scaffold both cognitive–cultural integration and emotional–social adjustment by leveraging distinct environmental affordances.

## 5. Discussion

This study set out to explore how study tours facilitate international students’ cross-cultural adaptation. Building on a constructivist grounded theory approach, our findings illuminate a process through which students transition from initial cultural distance to multidimensional adaptation, shaped by context-specific engagements, situational behaviors, and emotional responses in study tours. By foregrounding the role of environmental setting, the study shows how different context settings affect subsequent behavior, emotions, and cross-cultural adaptation. This process was shown to unfold dynamically across three phases—pre-departure, on-site immersion, and post-tour reflection—highlighting the embodied and situational nature of adaptation. The study contributes to a deeper understanding of how adaptation occurs not only as an individual psychological shift but also as a context-mediated and socially constructed process.

### 5.1. Cross-Cultural Distance as Pre-Embodied Constraints on Adaptation

The study identifies four interrelated dimensions of cross-cultural distance: linguistic barriers, cultural living practices, psychological resistance, and interpersonal gaps, which structure students’ pre-tour orientation toward the host culture. Instead of isolated obstacles, we frame these as pre-embodied constraints that affectively and perceptually internalized dispositions formed during early experiences abroad.

Linguistic barriers, echoing Young and Schartner (2014), go beyond communication and impact students’ sense of agency and belonging in academic and social spaces. Cultural living practice—linked to routines, food, or etiquette—mirrors Cheung et al.’s (2021) and Lin et al.’s (2022) accounts of disrupted bodily routines and generated cultural fatigue. Psychological resistance, including emotional hesitation and third-person detachment (e.g., referring to locals as “they”), aligns with Cao and Meng’s (2022) view of vulnerability during intercultural transition. As Dorsett et al. (2019) note, interpersonal gaps reflect a lack of contact and less social cognition that can hinder integration.

Thus, these four dimensions reflect a common experiential state that international students develop during their initial academic and social exposure in the host country. Identifying this state provides a meaningful baseline to observe and compare the effects of study tours as emotionally and environmentally situated interventions.

### 5.2. Study Tours as Situated, Embodied Process Variables in Cross-Cultural Adaptation

This study conceptualizes study tours not simply as discrete experiences, but as situated and embodied process variables that actively mediate international students’ cross-cultural adaptation. Rather than treating adaptation as a static trait or linear outcome, we view it as a dynamic, co-constructed process shaped by students’ bodily engagement with diverse sociocultural environments over time. This framing is made possible through our use of CGT, which allows for the emergence of processual insights grounded in participants’ lived experience.

By integrating the situated learning theory (Lave & Wenger-Trayner, 1991/2011) and embodied cognition theory (Wilson & Beard, 2013), we highlight the mechanisms through which study tours act as catalysts for adaptation. In contrast to routine academic settings—often abstract and cognitively detached—study tours offer authentic contexts where knowledge is co-constructed through physical actions, emotional responses, and meaningful interaction with people and places.

Specifically, situated learning emphasizes active engagement in authentic environments. Our data show that students participated in physically grounded cultural activities (e.g., ink-making, kung fu, climbing) and formed contextual interactions with peers and locals, generating emotions like pride, calmness, and relaxation. This sequence aligns with Riggan et al. (2011) and Viwatronnakit et al. (2019), who argue that perception, action, and emotion are deeply interlinked, and bodily states can shape meaning-making.

From an embodied cognition perspective, adaptation is both cognitive and sensorimotor. Students’ physical engagement, sensory immersion, cognitive exchange, and social bonding generate emotion that jointly mediate how cultural meanings are internalized (Foglia & Wilson, 2013; Schwarz, 2013; He & Chen, 2023; Li et al., 2024).

Furthermore, the study differentiates culture-led and nature-led settings in shaping different embodied experiences. Culture-led settings (e.g., guided museum visits or heritage workshops) are structured and symbolically rich, fostering cognitive participation (e.g., reflection or skill learning) and evoking cognitive emotions like curiosity and pride, while nature-led environments (mountains, beaches, and parks) serve as affective and sensory spaces, supporting affective decompression and relaxation through informal, sensory-rich interactions (e.g., hearing the ocean waves or yelling from the mountaintop). These findings build on Leung et al. (2011), suggesting that the sensory-emotional qualities of specific environments shape adaptation.

Thus, study tours should not be seen as mere cultural exposure tools. Instead, they function as dynamic process variables that initiate embodied, emotionally charged, and socially situated learning episodes, facilitating transformation in how students perceive, feel, and act within unfamiliar cultural landscapes.

### 5.3. Differentiated Impacts of Cultural- and Nature-Led Settings on Cross-Cultural Adaptation

Building on the study tours’ embodied and situated nature, our findings suggest that culture- and nature-led environments shape cross-cultural adaptation through distinct mechanisms. The culture-led setting experiences facilitate deeper engagement with host values and promote cultural inclusion, critical reflection, and identity reconstruction, which are key dimensions of cultural integration (Chan & Klayklueng, 2018; Nelson & Luetz, 2021). In contrast, the nature-led setting experiences are more efficient in social and psychological dimensions, such as stress reduction, emotional resilience, and social participation and network formation. This supports findings by Buckley (2020) and Juliana et al. (2024) on the therapeutic and relational benefits of nature immersion. Thus, we propose that nature-led environments tend to function as “emotional stabilizers” that facilitate social integration and psychological adjustment. In contrast, culture-led settings are more effective as “cognitive integrators”, which are more efficient in promoting cultural integration, such as cultural meaning and identity construction.

Moreover, our study further differentiates three progressive levels within each dimension of adaptation; for example, social integration includes social inclusion, participation, and network formation. International students experience these levels to varying degrees, which to some extent reflect differences in the degree of adaptation achieved by students. Such variation may be shaped by factors such as context-person fit, depth of situational immersion, and prior intercultural experience.

## 6. Conclusions

This study adopts a constructivist grounded theory approach and draws on the situated learning theory (Lave & Wenger-Trayner, 1991/2011) to conceptualize cross-cultural adaptation as a dynamic process co-constructed through international students’ ongoing interactions with others, environments, and meaning-making contexts. Within this framework, study tours are understood as embodied, situated, and environment-sensitive sites that accelerate the transition from cross-cultural distance to psychological, social, and cultural integration. By incorporating the embodied cognition theory (Wilson & Beard, 2013), the analysis further highlights how sensory and affective experiences serve as key triggers for adaptation. Guided by Porth’s (1997) three-phase model, the study constructs a processual framework that traces how context-specific engagements during the pre-departure, on-site, and post-tour stages collectively shape the adaptive trajectory. This framework underscores the role of space and environment in mediating intercultural learning and sets the stage for rethinking study tour design through a more contextualized and experiential lens. In doing so, the study addresses the theoretical gap around processual and experiential dimensions of cross-cultural adaptation and underscores the value of study tours as intentional, context-sensitive mechanisms for fostering meaningful intercultural learning.

### 6.1. Theoretical Contributions

First, this study advances a process-oriented, context-sensitive understanding of cross-cultural adaptation by clarifying how study tours facilitate adaptation through embodied, affective, and environmental mechanisms. It conceptualizes study tours as embodied, situated, and environment-sensitive learning spaces, extending the situated learning and embodied cognition theories to the context of short-term intercultural education. We affirm that such programs accelerate psychological, social, and cultural adaptation via intensive social interaction, activity-based engagement, and context-specific experiences.

Second, the study extends the traditional acculturation model (Berry, 1997; Searle & Ward, 1990) by identifying cultural integration as a distinct adaptation dimension. Unlike psychological or sociocultural adjustment, cultural integration involves identification with, reinterpreting, and proactively disseminating host culture values, signaling a deeper, developmentally advanced form of adaptation with broader intercultural implications.

Third, this study unpacks each domain of cross-cultural adaptation into internally progressive levels. This layered conceptualization highlights adaptation as a gradual, accumulative process, offering a scalable framework to evaluate the depth of intercultural development and a more nuanced understanding of how international students evolve through different stages of engagement and identification.

### 6.2. Practical Implications

The study offers several actionable strategies for educators and program designers seeking to maximize the adaptive potential of study tours, particularly by aligning program design with students’ evolving psychological, social, and cultural needs.

First, organizers should clearly distinguish between culture-led and nature-led activities when designing study tours. By understanding their differential functions, these two types of settings should be intentionally combined based on their unique affordances, enabling more purposeful program design.

Second, study tours should be structured around students’ adaptive readiness, recognizing that emotional and social needs vary across individuals and over time. Tour organizers can introduce nature-led experiences early in the itinerary to reduce emotional resistance and build relational safety, while progressively integrating more cognitively demanding, culture-led activities as students become more emotionally settled. This sequencing ensures that participation intensity aligns with students’ emotional readiness, sustaining engagement, and supporting adaptive progression.

Third, ongoing process support mechanisms should be embedded into tour design. Tools like reflective journaling, peer-sharing, or digital mood tracking can help students articulate emotional responses and monitor adaptation progress. These indicators enable timely pedagogical adjustments—such as shifting toward restorative activities during stress peaks—allowing study tours to function as adaptive systems responsive to students’ psychological rhythms.

### 6.3. Limitations and Future Research

This study has several limitations that should be acknowledged and addressed in future research. First, the study did not systematically distinguish students by their prior intercultural exposure—including variations in the length of stay in China—which may influence their engagement levels and adaptation outcomes. While such variation introduced potential bias, it also offered analytical value by enabling the identification of different stages or depths of adaptation. Future research could classify students more systematically based on intercultural experience to trace segmented trajectories and enhance contextual interpretation.

Second, the research was conducted solely within a Chinese cultural setting. While this offers insight into East Asian study tour contexts, the findings may not generalize to other cultural or institutional environments. Cross-contextual validation is needed to extend the framework’s applicability.

Third, although a processual framework was developed, the study used a cross-sectional design and cannot track adaptation over time. Longitudinal approaches—such as follow-up interviews, diary studies, or experience sampling—could better capture how identity, emotional resilience, and cultural integration evolve across and beyond the study tour experience.

## Figures and Tables

**Table 1:** Basic information of interview participants.

Participant ID	Nationality	Ethnic Background	Length of Stay in China	Language Ability	Purpose for Study
V1	Thailand	Thai	3 years	High	Undergraduate in Business
V2	Malaysia	Malaysian Chinese	2 years	Intermediate (HSK 4)	Bachelor’s in Chinese Culture
V3	Indonesia	Javanese	8 months	Basic	Short-term Exchange
V4	Laos	Lao	1 year	Basic	Bachelor’s in Fine Arts
V5	Thailand	Thai	1 year	Intermediate	Chinese Language Training
V6	Malaysia	Malaysian Chinese	6 months	Basic	Pre-University Language Preparation

**Table 2:** Examples of initial coding from raw data.

Data Snippet	Codes	Initial Concepts	Labels	Memos	Source
*“It was my first time to study abroad … I couldn’t communicate well with others …I didn’t speak English or Chinese well.”*	First time abroad; could not communicate; poor Chinese and English	difficulty in expression, lack of communication skills; feeling of strangeness	Early-stage language barrier	The overlap of language barriers and first-time study abroad may imply emotional tension or unease, even though no explicit emotions are expressed. This warrants further exploration in the emotional dimension.	Diaries
*“We went to Gulangyu Island… visited the Pie Museum…local people taught us learned how to make pie…I made a video and want to share* *the process with my good friends* *.”*	Cultural visit; food-making; interaction with local people; sharing cultural experiences	hands-on learning from locals; desire to share the host culture	Cultural participation; cultural storytelling	Cultural participation and social interaction often co-occur; it is worth examining whether this constitutes a recurring interaction pattern. Students show more signs of cultural communication when participating in cultural activities.	
*“We went to Wuyi Mountain… climbed a mountain… bamboo rafting… the scenery was beautiful… relaxing… made a friend…we are still good friends now.”*	Mountain climbing; bamboo rafting; scenic appreciation; relaxation; making friends	natural site visit; physical participation; emotional relief building lasting intercultural friendships	Natural immersion; physical engagement; social bonding	Frequent mentions of nature and relaxation appear to be closely linked to bodily sensations.	Interview

**Table 3:** Core dimensions and constituent elements of cross-cultural adaptation during study tours.

Main Categories		Sub-Categories
Cross-cultural Distance		Linguistic Barriers
		Cultural Living Practices
		Psychological Resistance
		Interpersonal gaps
Study Tour Context	Environment Settings	Culture-led Settings
		Nature-led Settings
	Situational Behaviors	Physical Engagement
		Sensory immersion
		Cognitive Exchange
		Social Bonding
	Short-term Emotions	Cognitive Emotions
		Affective State
Cross-culture Adaptation	Cultural Integration	Cultural Understanding
		Cultural Identity
		Cultural Dissemination
	Social Integration	Social Inclusion
		Social Participation
		Social Network Formation
	Psychological Adjustment	Emotional Stability
		Stress Reduction
		Emotional Resilience

## Data Availability

Please contact the first author for access to the research data.

## Appendix A. Semi-Structured Interview Guide: Exploring Cross-Cultural Adaptation Through Study Tours

**Section A: Basic Information and Background**

- Can you briefly introduce yourself? (Nationality, academic program, length of stay in China.)
- When did you join the most recent study tour?

**Section B: On-Site Experiences during the Study Tour**

- Please describe the destination of your study tour and the main attractions you visited.
- What do you remember most vividly from the study tour? Why?
- Were there any cultural attractions (museums, factories, etc.) in the tour?
  ■ How did you feel during or after these activities?
- Can you describe any activities that involved hands-on participation (e.g., craft making, physical tasks)?
  ■ How did you feel during or after these activities?
- Were there any natural attractions (mountains, lakes, seaside, etc.) in the tour?
  ■ How did you feel during or after these activities?
- Did you have any meaningful conversations or exchanges during the tour?
- With whom? What did you learn or feel?

**Section C: Emotional Reflections**

- Were there any moments when you felt curious, proud, surprised, or emotionally touched?
- What triggered these emotions?
- Did you feel more relaxed, joyful, or connected to others during certain parts of the tour?
- Can you describe the scenes where this occurred?

**Section D: Post-Tour Impact and Adaptation**

- After the tour, did anything change in how you see or understand Chinese culture?
- Did the experience influence your social relationships with peers or local people?
- Do you think the study tour helped you become more emotionally comfortable or confident living in China?
- If you compare your experience before and after the tour, do you feel any changes in your mindset, behavior, or cultural openness?
- In your opinion, what aspects of the tour had the most lasting influence?

**Optional**

- Is there anything else you would like to share about your experience with the study tour and its impact on your life in China?
